# Impact of tooth brushing on oral bacteriota and health care-associated infections among ventilated COVID-19 patients: an intervention study

**DOI:** 10.1186/s13756-023-01218-y

**Published:** 2023-03-08

**Authors:** Iwona Gregorczyk-Maga, Anna Pałka, Mateusz Fiema, Michal Kania, Anna Kujawska, Paweł Maga, Estera Jachowicz-Matczak, Dorota Romaniszyn, Agnieszka Chmielarczyk, Barbara Żółtowska, Jadwiga Wójkowska-Mach

**Affiliations:** 1 Institute of Dentistry, Faculty of Medicine, Jagiellonian University Medical College, Ul. Montelupich 4, 31-155 Kraków, Poland; 2 Department of Endocrinology, University Hospital, Ul. Jakubowskiego 2, 30-688 Kraków, Poland; 3 Doctoral School of Medicine and Health Sciences, Jagiellonian University Medical College, Ul. św. Anny 12, 31-008 Kraków, Poland; 4 Chair of Metabolic Diseases, Faculty of Medicine, Jagiellonian University Medical College, Ul. Jakubowskiego 2, 30-688, Kraków, Poland; 5 Microbiology Unit, University Hospital, Ul. Jakubowskiego 2, 30-688 Kraków, Poland; 6 Chair of Angiology, Faculty of Medicine, Jagiellonian University Medical College, Ul. Jakubowskiego 2, 30-688, Kraków, Poland; 7 Chair of Microbiology, Faculty of Medicine, Jagiellonian University Medical College, Czysta 18, 31-121 Kraków, Poland; 8 Center for Innovative Therapy, Clinical Research Coordination Center, University Hospital, Ul. Jakubowskiego 2, 30-688 Kraków, Poland

**Keywords:** Oral microbiota, Dysbiosis, COVID-19, Mechanical ventilation, ARDS, VAP, HAI, Ventilator-associated pneumonia, Health care-associated infection

## Abstract

**Background:**

Up to 48% of ventilated coronavirus disease 2019 (COVID-19) patients develop ventilator-associated pneumonia (VAP) during hospitalization in an ICU. Dysbiotic oral microbiota can colonize the lower respiratory tract and lead to VAP. It is recommended to introduce oral care strategies in the ICU to prevent VAP. In this study, we observed the impact of an oral hygienic protocol with tooth brushing on cultivable oral bacteriota, the incidence of HAI and patient safety among mechanically ventilated COVID-19 patients in an ICU setting.

**Methods:**

In this prospective cohort study, we recruited 56 adult COVID-19 patients who qualified for mechanical ventilation. Patients were divided into 2 groups depending on the oral care procedure: standard and extended oral procedures with tooth brushing. Oral bacteriota samples were taken first within 36 h and after 7 days of intubation. Microorganisms were identified by MALDI/TOF mass spectrometry. bacterial health care-associated infection (HAI) cases were retrospectively analyzed by etiology. A PFGE study was performed for *Klebsiella pneumoniae* to check for clonal spreading of strains from oral bacteriota samples and HAI cases.

**Results:**

We observed significant dysbiosis and a decrease in cultivable oral bacteriota diversity, with a high frequency of potentially pathogenic species, including* Acinetobacter baumannii* and *K. pneumoniae*. The HAI incidence rate was high (55.2/1000 patient-days), most commonly of *K. pneumoniae* and *A. baumannii* etiologies, which correlated with the presence of *A. baumannii* and *K. pneumoniae* in the oral samples. Strains isolated from VAP cases were the same as oral isolates in 8 cases. The procedure with tooth brushing led to less frequent identification of *A. baumannii* in oral samples (55.6% vs. 5.3%, *p* = 0.001); however, it did not decrease the incidence of HAIs.

**Conclusions:**

Dysbiotic oral bacteriota is an important source of respiratory pathogens. The introduction of tooth brushing in oral hygiene protocols in an ICU setting was effective in decreasing the extent of oral bacteriota dysbiosis; however, it did not reduce the risk of HAIs or mortality.

*Trial registration*: 1072.6120.333.2020.

**Supplementary Information:**

The online version contains supplementary material available at 10.1186/s13756-023-01218-y.

## Introduction

Interactions between the human oral microbiota and severe acute respiratory syndrome coronavirus 2 (SARS-CoV-2) are currently being intensively investigated [1]. SARS-CoV-2, similar to other coronaviruses, replicates in the oral cavity [2] and can cause pneumonia. It is estimated that approximately 15–20% of coronavirus disease 2019 (COVID-19) patients require admission to the intensive care unit (ICU) due to acute respiratory distress syndrome (ARDS). This population is at high risk of developing bacterial or fungal coinfections, with previous reports showing a high incidence of *Enterobacter* spp*., Acinetobacter baumannii* and *Aspergillus* spp. [3–5], health care-associated infections (HAIs). Up to 48% of ventilated COVID-19 patients develop ventilator-associated pneumonia (VAP) during hospitalization in an ICU, as reported in a recent meta-analysis [6]. VAP is a known risk factor for poor outcomes in this population of patients [7]; thus, it is important to identify and introduce effective interventions to prevent VAP. It is crucial in our setting, as studies have shown that HAIs are a major concern in Poland, and we lack proper monitoring [8–10].

Dysbiotic oral microbiota has the potential to colonize the lower respiratory tract [11] and lead to severe complications, including VAP. Oral aspiration is a key factor leading to lower respiratory tract infections.

There is a range of actions that can lower the risk of contamination of the lower respiratory tract during intubation and limit the occurrence of VAP [12, 13], but few have been investigated in larger trials. Avoiding intubation is recommended, as it has a major impact on the incidence of VAP; however, in many cases, there is no other treatment option. The modification of endotracheal tube cuff shapes or materials enabled minimization of fluid seepage into the lungs [14, 15]. Proper oral hygiene protocols were proven to limit the incidence of VAP in an ICU setting [16]. Nevertheless, the evidence for specific interventions is lacking or even conflicting.

The latest update of the European Respiratory Society (ERS), European Society of Intensive Care Medicine (ESICM), European Society of Clinical Microbiology and Infectious Diseases (ESCMID) and Asociación Latinoamericana del Tórax (ALAT) guidelines [17] recommended against the use of chlorhexidine as a selective oral decontamination for patients requiring mechanical ventilation, as some studies revealed that exposure to chlorhexidine oral care was associated with an increased risk of death [18].

In a recent Cochrane Library systematic review on oral care strategies for critically ill patients to prevent VAP [19], the authors concluded that antiseptics and tooth brushing may be more effective than standard oral health hygiene strategies in reducing the incidence of VAP and the length of ICU stay.

It is recommended to introduce oral care strategies as part of the medical treatment plan when a patient is admitted to the ICU [20]. Nevertheless, the practice of tooth brushing is not universally incorporated in local oral health protocols. In Poland, there is no clear consensus of the recommended oral health care protocols in ICUs.

The aims of this study were as follows:

1. Observation of dynamics in cultivable oral bacteriota among mechanically ventilated COVID-19 patients in an ICU setting
2. Evaluation of the incidence of HAIs and VAP and their association with cultivable oral bacteriota among mechanically ventilated COVID-19 patients in an ICU setting
3. Assessment of the impact of different oral hygienic procedures on cultivable oral bacteriota, the incidence of HAI and patient safety among mechanically ventilated COVID-19 patients in an ICU setting and approaches to oral care in an ICU setting

## Materials and methods

### Study design and participants

Recruitment for this prospective cohort study took place in the University Hospital in Krakow, Poland. Patients were offered the opportunity to participate in the study and asked for the signed consent form on admission to the hospital. During hospitalization, 56 of them qualified for intubation and were hospitalized in the temporary intensive care unit (ICU) for COVID-19 patients between 1st September and 31st January 2022.

The inclusion criteria were as follows:

1. SARS-CoV-2 infection confirmed by real-time reverse transcriptase-polymerase chain reaction (RT‒PCR) assay of nasal and pharyngeal swabs upon hospital admission
2. Signed consent to participate in the study
3. Patients admitted to the ICU
4. Intubation due to COVID-19-related pneumonia and acute respiratory distress syndrome (ARDS) within 36 h preceding study procedures

All medical procedures in the ICU were performed according to the local standards. To prevent leakage of fluids below the laryngeal/tracheal cuff, VBM medical manometers 21 [21] were used. The cuff pressure was measured by the nursing staff 3 times/day, with a normal range between 40 and 60 mmHg.

Demographic and clinical data were gathered from the hospital electronic medical records, including but not limited to age, sex, date of COVID-19 diagnosis, admission to the hospital and ICU, date of intubation, selected comorbidities, pre- and postintubation treatment, including systemic steroids and antibiotics, days of antibiotic treatment (DOT) before and after intubation (the number of Days a patient receives an antibiotic). Selected baseline and maximal laboratory results were also extracted.

### Oral health assessment

On admission to the ICU, the patients’ oral health was assessed utilizing the modified Beck Oral Assessment Score (BOAS), consisting of 5 subscales: assessment of lips, mucosa and gingiva, tongue, teeth, and saliva. A higher score reflects dysfunction or tissue injury. BOAS scores range from 5 (no oral dysfunction) to 20 (severe dysfunction). A score greater than 5 is abnormal [22, 23] (Additional file 1: Appendix A1).

### Oral cavity sampling methods

Samples were taken two times, first within 36 h of intubation (baseline) and again after 7 days of intubation (follow-up).

In each sampling, four oral habitats were sampled: buccal mucosa, tongue, buccal dental surface and gingival pocket. Every sample was taken by a trained dentist.

Sampling the posterior part of the dorsum of the tongue and buccal mucosa, we used ESwab™. ESwab combines a COPAN-invented flocked swab with 1 mL of liquid amine in a plastic, screw cap tube. Dental plaque was collected from the buccal dental surface side using Tooth Cleanic KerrHawe—KWX-OP-SZ-011, and after collection, the brush was placed in 1 mL of Liquid Amies in a plastic screw cap tube. Three pieces of PerioPaper Strips, designed to absorb or carry 0–1.2 µl of fluid, were used to collect gingival cerficular fluid (GCF) samples. The strips were placed in the gingival pocket for 30–45 s until its surface soaked up. To minimize the risk of preanalytical errors during sample collection, sterile gauze was used to remove excess saliva from the mucosae and dry the dental surfaces, preventing salivary contamination of GCF.

### Intervention

Patients were divided into 2 groups depending on the oral care procedure:

1. Standard oral procedure (SP, cleaning and moisturizing of oral cavity, suction of excess fluid)
2. Extended oral procedure with tooth brushing (EP, cleaning and moisturizing of the oral cavity, tooth brushing, suction of excess fluid)

The procedures were performed twice daily in each patient. Detailed information on these procedures is included in Additional file 1: Appendix A2. The allocation protocol was based on the patients’ location in the ward. Each room was assigned one type of procedure (standard—even number or extended—uneven number of rooms) that was permanent during the study period. The efficacy of two different procedures for oral care in an ICU setting was assessed.

### Microbiological cultures

After the collection of patient samples, they were delivered as soon as possible to the microbiological laboratory for the purpose of microorganism isolation. Banking and identification of bacterial strains from HAIs of all hospitalized patients (samples were collected throughout the duration of the whole hospitalization, not only during the intervention) in the temporary units for COVID-10 patients in the UH were performed in the Department of Microbiology, UH, Krakow. Various diagnostic samples were collected, cultured and assessed for the microbial etiology of infections. In this group, 11 K*. pneumoniae* strains from HAIs of patients under surveillance (with SP or EP) were used for further analysis: 5 from PNU, 3 from BSI and 3 from UTI cases. One strain from an oral sample of patient 6 was not stored. Strains from oral samples and HAIs were stored in the Department of Microbiology, UH, Krakow, at − 70 °C using Microbank^®^ (Biomaxima, Lublin). Dilutions of samples (from − 1 to − 6) were inoculated on the following media: McConkey (Graso, Biotech), Columbia (Lab-Agar, Biomaxima), Scheadler (Scheadler-Agra, Biomaxima), Bile Esculine Azide (Lab-Agar, Biomaxima), and MRS Agar (Oxoid). Simultaneously, swab samples were inoculated with the qualitative culture method using the same media as in the dilution method. Media were aerobically incubated at 37 °C for 24 h (McConkey, Columbia, Bile Esculine Azide) or anaerobically at 37 °C for 48 h (M.R. S and Scheadler, GENbag Atmosphere Generators, BioMérieux, France). Next, the colonies were counted and phenotypically reported, and the results are presented as CFU/mL (colony forming unit). The microorganisms were identified using MALDI TOF MS mass spectrometry (Vitek MS Home bioMérieux).

### Laboratory-confirmed health care-associated infections

The bacterial health care-associated infection (HAI) cases were analyzed retrospectively using definitions from the Healthcare-Associated Infections Surveillance Network (HAI-Net) [24], including bloodstream infections (BSI), pneumonias (PNU), urinary tract infections (UTI) and others. The cases and samples were obtained via passive surveillance defined as identifying and reporting of HAIs by nontrained personnel. In 2020–2022, no active, prospective surveillance and registration of HAIs was carried out in the hospital; therefore, there was no other source of information on HAIs that had not been microbiologically confirmed. LOS was defined as the sum of the days of stay (patient days, pds), including the day of admission and the day of discharge.

Microbiological samples were routinely acquired when there was suspicion of infection by taking blood, bronchoalveolar lavage (BAL) or urine samples. Only laboratory-confirmed cases based on culture growth qualified for the analysis; with multiple samples from the same infection case, only the first isolate from each patient was selected for microbiological analysis, with subsequent patient and HAI cultures excluded.

Multiple logistic regression models were built to identify predictors of HAI of *A. baumannii, Enterococcus faecalis* and *Klebsiella. pneumoniae* etiology (3 etiology-specific models) that included the type of oral care procedure applied, identification of *A. baumannii/E. faecalis/K. pneumoniae* in the baseline oral sampling, baseline BOAS score category and pre-ICU antibiotic use.

### Pulsed-field gel electrophoresis (PFGE)

All isolates of *Klebsiella pneumoniae* were analyzed using the standardized PFGE protocol developed at the Centers for Disease Control and Prevention by the PulseNet program (version for *Escherichia coli*). Data from previous studies showed that *K. pneumoniae* strains isolated in Polish hospitals (mainly from ICUs for adults and neonates and pediatric units) often belong to one clone. For this reason, a PFGE study was performed for this species to check for clonal spreading of strains coming from oral bacteriota samples and HAI cases. Genomic DNA was prepared in situ in agarose blocks and was subsequently digested with restriction enzymes: XbaI (25 U per block, Thermo Scientific. The digested products were separated on a CHEF III PFGE system (Bio-Rad, Warsaw, Poland) in 0.5 × Tris-borate-EDTA buffer at 14 °C at 6 V for 22 h with a starting pulse of 2 s and a final pulse of 35 s. GelCompar (Applied Maths, Kortrijk, Belgium) was used for cluster analysis with the unweighted pair group method with an arithmetic mean and the Dice coefficient. Similarity must be > 90% for the pattern to be considered to belong to the same pulsotype. The results of the PFGE analysis are presented for each patient with a number that they were assigned on recruitment (1–56). The term “case” refers to HAI diagnoses.

### Ethics statement

The study and its protocol were approved by the Jagiellonian University Bioethics Committee, decision number 1072.6120.333.2020; Dec 7, 2020, and 1072.6120.353.2020. Dec 16, 2020. Written informed consent was obtained from each subject prior to participation.

### Statistical analysis

The PS Imago Pro ver. 7.0 was used for all statistical analyses. The normality of the continuous variable distribution was assessed using the Shapiro‒Wilk test. Differences between groups were analyzed with Student’s t test or nonparametric test (Mann–Whitney U test) when appropriate. Paired data were analyzed using the Wilcoxon test. Continuous variables are presented as the arithmetic mean (x̄) ± standard deviation (SD) or as the median with interquartile range (IQR) when the data were not normally distributed. The distribution of categorical variables was described as counts and percentages. Statistical testing was completed to compare categorical variables using an independent sample chi-squared test or Fisher's exact test when appropriate and dependent samples with McNemar’s test. We built a multivariate logistic regression model including an arbitrarily chosen number of predictors. We assured that the multicollinearity assumption was fulfilled. Negelkerke’s index was used as the coefficient of determination, R2. We measured the strength association by the odds ratio (OR) and 95% confidence intervals (CI). Statistical inference was set at *p* < 0.05.

## Results

### Demographic and clinical characteristics

The study population included 56 patients admitted to an ICU who required mechanical ventilation due to COVID-19-related ARDS. The mean age of the participants was 66.5 ± 12.7 years, and there were 24 (42.9%) females. The population was obese with a mean BMI of 31.9 ± 5.8. Sixteen (28.6%) were admitted directly from the emergency department, and 40 (71.4%) were transferred from another hospital ward. The mean time from COVID-19 diagnosis to intubation was 6.95 ± 6.62 days. Pre-ICU, systemic steroid therapy was used in 76.9% and antibiotics in 63.5% of patients. The median antibiotic days of therapy (DOT) before intubation was 7 (IQR 3–13.5). The full pre-ICU characteristics of the study population have been previously described elsewhere [25].

In the ICU, steroids and antibiotics were used in 85.7% and 55.4% of patients, respectively, with a median DOT of 8 (IQR 4–14) after intubation. The full baseline and follow-up characteristics of the study participants are presented in Table 1.

**Table 1:** Baseline comparison of participants according to procedure

Characteristics	Standard procedure N = 25	Extended procedure with tooth brushing N = 31	*p* value
Age [years]	67 (56–73.5)	67 (62–79)	NS
Female [n (%)]	10 (40.0%)	14 (45.2%)	NS
WHO ordinal scale, on admission to an ICU [43]	7 (6–9)	6 (5–9)	NS
Baseline BOAS, sum score	11 (9.5–12.5)	12 (11.25–14.8)	0.032
*Baseline BOAS category*			NS
1–10	9 (36.0%)	4 (16.7%)	
11–20	16 (64.0%)	20 (83.3%)	
Baseline BOAS, teeth	3 (2–3)	3 (2–4)	NS
*In-hospital pharmacotherapy before intubation [n (%)]*			
Steroid therapy	19 (76.0%)	21 (77.8%)	NS
Antibiotic	19 (76.0%)	14 (51.9%)	NS
DOT before intubation [days]	7 (2.8–13.3)	7 (3–15)	NS
Baseline number of genera, all sites	4 (3–5)	5 (4–6)	0.031
Baseline number of species, all sites	5 (4–6)	7 (5–9)	0.006
*Baseline number of patients with selected genera/species*			
*Acinetobacter baumannii*	31.0%	16.1%	NS
*Enterococcus faecalis*	52.0%	29.0%	NS
*Escherichia coli*	16.0%	19.4%	NS
*Klebsiella pneumoniae*	28.0%	12.9%	NS
*Lactobacillus* spp.	56.0%	58.1%	NS
*Streptococcus* spp.	76.0%	83.9%	NS
*Baseline CFU/ml from oral samples*			
All bacterial strains	4.0E + 5 (5.0E + 4–1.5E + 6)	4.0E + 5 (1.0E + 5–1.4E + 6)	NS
All G + strains	5.0E + 5 (1.5E + 5–1.5E + 6)	5.0E + 5 (1.5E + 5–1.5E + 6)	NS
All G- strains	2.3E + 4 (2.8E + 3–2.3E + 5)	2.0E + 5 (2.0E + 4–8.0E + 5)	0.021
*Streptococcus* spp.	7.0E + 5 (1.8E + 5–2.2E + 6)	4.0E + 5 (1.5E + 5–1.5E + 6)	NS
*Lactobacillus* spp.	1.0E + 6 (2.0E + 5–2.0E + 6)	9.0E + 5 (2.0E + 5–2.0E + 6)	NS
*Klebsiella pneumoniae*	3.0E + 4 (3.0E + 2–5.0E + 4)	1.5E + 4 (1.6E + 3–1.4E + 5)	NS
*Acinetobacter baumannii*	1.3E + 4 (2.3E + 3–1.9E + 4)	1.0E + 4 (1.6E + 3–1.1E + 5)	NS
*Enterococcus faecalis*	3.0E + 5 (7.0E + 4–1.0E + 6)	5.0E + 5 (2.1E + 4–5.4E + 6)	NS

Data are presented as the means (SDs), medians (Q1–Q3) or N [%]

*WHO*, World Health Organization; *ICU*, intensive care unit; *BOAS*, Beck Oral Assessment Scale, *DOT*, days of antibiotic therapy, *HAI*, health care-associated infection, *CFU*, colony forming unit, *NS*, not significant

### Oral cultivable bacteriota composition

Between the baseline and follow-up oral sampling, the number of bacterial genera did not change, but a decrease in the overall number of species from all sites was observed (median 6 vs. 4, *p* = 0.005). Specifically, the number of species in mucosal swabs, dental surface samples and the tongue were lower at follow-up (medians 3, 4, and 3 vs. 2, 3, and 2; *p* = 0.008, *p* = 0.04 and *p* = 0.17, respectively). There were no differences in the overall CFU counts of all bacterial strains from all sites between baseline and follow-up.

At baseline, the number of patients with *E. faecalis, A. baumannii, K. pneumoniae* and *E. coli* in oral samples was high—39.3%, 23.2%, 19.6% and 17.9%, respectively. There was a general decrease in the rate of identification of all species at follow-up, but it remained relatively high for *E. faecalis* and *A. baumannii*—both 29.7%.

The complete qualitative and quantitative bacteriota characteristics are presented in Tables 2 and 3.

**Table 2:** Follow-up comparison of participants according to procedure

Characteristics	Standard procedure N = 18*	Extended procedure with tooth brushing N = 19*	*p* value
Follow-up BOAS, sum score	11.5 (9–14.5)	11 (9–13)	NS
*Baseline BOAS category*			NS
1–10	8 (44.4%)	6 (40.0%)	
11–20	10 (55.6%)	9 (60.0%)	
Follow-up BOAS, teeth	3 (2–4)	2 (1–3)	0.026
Follow-up number of genera, all sites	4 (2.8–5.3)	3 (2–4)	NS
Follow-up number of species, all sites	4.5 (4–6.3)	3 (2–5)	NS
*Follow-up number of patients with selected genera/species*			
*Acinetobacter baumannii*	55.6%	5.3%	0.001
*Enterococcus faecalis*	55.6%	5.3%	0.001
*Escherichia coli*	11.1%	5.3%	NS
*Klebsiella pneumoniae*	16.7%	15.8%	NS
*Lactobacillus* spp.	27.8%	47.4%	NS
*Streptococcus* spp.	61.1%	42.1%	NS
*Follow-up CFU from oral samples*			
All bacterial strains	3.3E + 5 (5.8E + 4–1.1E + 6)	1.9E + 5 (5.0E + 4–4.8e + 5	0.067
All G + strains	8.0E + 5 (2.0E + 5–1.7E + 6)	1.4E + 5 (4.3E + 4–3.0E + 5)	< 0.001
All G- strains	3.0E + 4 (2.6E + 3–3.1E + 5)	7.5E + 5 (1.1E + 5–1.9E + 6)	0.004
*Streptococcus* spp.	7.5E + 5 (3.0E + 5–1.0E + 6)	8.5E + 4 (3.5E + 4–5.3E + 5)	0.008
*Lactobacillus* spp.	1.0E + 6 (1.5E + 5–2.0E + 6)	1.5E + 5 (8.0E + 4–3.0E + 5)	0.039
*Klebsiella pneumoniae*	1.2E + 5 (6.5E + 2–1.1E + 7)	3.5E + 5 (1.4E + 5–4.8E + 5)	NS
*Acinetobacter baumannii*	3.5E + 4 (4.8E + 3–3.1E + 5)	3.0E + 4 (2.0E + 4 – NA)	NS
*Enterococcus faecalis*	NA	NA	NA

Data are presented as the means (SDs), medians (Q1–Q3) or N [%]

*WHO*, World Health Organization; *ICU*, intensive care unit; *BOAS*, beck oral assessment scale, *DOT*, days of antibiotic therapy, *HAI*, health care-associated infection, *CFU*, colony forming unit, *NS*, not significant, *NA*, not available

*Number of patients who survived until the follow-up

**Table 3:** Baseline and follow-up characteristics by procedure performed

Characteristics	Standard procedure			Extended procedure with tooth brushing		
	Baseline	Follow-up	*p* value*	Baseline	Follow-up	*p* value*
BOAS, sum score	11 (9.5–12.5)	11.5 (9–14.5)	NS	12 (11.25–14.8)	11 (9–13)	NS
*BOAS category*						
1–10	9 (36.0%)	8 (44.4%)	NS	4 (16.7%)	6 (40.0%)	NS
11–20	16 (64.0%)	10 (55.6%)	NS	20 (83.3%)	9 (60.0%)	NS
BOAS, teeth	3 (2–3)	2.5 (2–3)	NS	3 (2–4)	2 (1–3)	0.026
*Percentage of patients with selected genera/species in oral samples*						
*Acinetobacter baumannii*	31.0%	55.6%	NS	16.1%	5.3%	NS
*Enterococcus faecalis*	52.0%	55.6%	NS	29.0%	5.3%	NS
*Escherichia coli*	16.0%	11.1%	NS	19.4%	5.3%	NS
*Klebsiella pneumoniae*	28.0%	16.7%	NS	12.9%	15.8%	NS
*Lactobacillus spp.*	56.0%	27.8%	NS	58.1%	47.4%	NS
*Streptococcus spp.*	76.0%	61.1%	NS	83.9%	42.1%	0.039

Data are presented as the means (SDs), medians (Q1–Q3) or N [%]

*BOAS*, beck oral assessment scale, *NS*, not significant

*p value for comparison baseline versus follow-up

### Impact of the extended oral care procedure with tooth brushing

The extended procedure with tooth brushing was performed for 55.4% of patients. Oral samples were taken from 56 patients at baseline and 37 (66.1%) 7 days post intubation due to death before the prespecified follow-up sample collection date. The 7-day mortality rate in our study sample was 33.9%.

There were some significant baseline differences between the intervention subgroups, with patients undergoing EP in more severe condition and with worse baseline oral health status (Table 1).

At follow-up, there were no differences in the total BOAS score, mortality rate or hospital infection rate. Patients who had EP performed had significantly lower follow-up BOAS teeth subscale scores after 7 days of observation (median 2 [IQR 1–3] vs. 3 [IQR 2–4], *p* = 0.026).

EP resulted in a lower occurrence of *E. faecalis* and *A. baumannii* compared to the standard procedure after 7 days of intubation (both 55.6% vs. 5.3%, *p* = 0.001). The overall CFU of all bacterial strains from all sites was lower in the EP group, but it did not reach statistical significance (median 3.3E + 5 [IQR 5.8E + 4–1.1E + 6] vs. 1.9E + 5 [IQR 5.0E + 4 = 4.8E + 5], *p* = 0.067).

The full comparison of baseline and follow-up characteristics of the study participants depending on the procedure performed is presented in Table 1.

### Bacterial health care-associated infections

In total, 52 different HAIs were detected, including 9 BSIs, 21 PNUs, and 22 UTIs. The HAI incidence density was 55.2/1000 pds. A total of 21.4% of patients had > 1 infection during hospitalization. There were no differences in the HAI incidence between the SP and EP groups (71.0% vs. 72.0%, *p* = 0.932). The most common etiologies for infections included *K. pneumoniae* (27.5% of patients with HAIs), *A. baumannii* (25%) and *E. faecalis* (25%). The polyetiological HAIs were confirmed in 25% of patients. There were no differences in HAI etiology between the oral care procedure groups. A summary of the cases of HAIs is presented in Table 4.

**Table 4:** Cases of infections in ventilated patients hospitalized in the ICU

	Standard procedure N = 25*, 436 patient-days		Extended procedure with tooth brushing N = 31*, 525 patient-days		*p* value
	Number of cases	Incidence rate	Number of cases	Incidence rate	
HAIs, any types of infections	24	55.1/1000 pds	28	53.3/1000 pds	NS
PNU	10	22.9/1000 pds	11	20.9/1000 pds	NS
BSI	4	9.2/1000 pds	5	9.5/1000 pds	NS
UTI	10	22.9/1000 pds	12	22.9/1000 pds	NS
*HAIs etiology*					
*Acinetobacter baumannii*	7	16.1/1000 pds	4	7.6/1000 pds	NS
*Enterococcus faecalis*	7	16.1/1000 pds	3	5.7/1000 pds	NS
*Klebsiella pneumoniae*	7	16.1/1000 pds	4	7.6/1000 pds	NS
Other	8	NA	21	NA	NA

*ICU*, intensive care unit, *HAI*, health care-associated infection, *VAP*, ventilator-associated pneumonia, *NS*, not significant, *NA*, not available

*Number of patients who were included in the standard/extended procedure, regardless of survival status

### Impact of oral cultivable bacteriota composition on health care-associated infections

There were some significant associations between selected species identified in the oral samples and HAIs. A higher incidence of VAP of any etiology was identified in patients with *A. baumannii* on follow-up sampling (100% vs. 42.9%, *p* = 0.023). The baseline and follow-up identification of *A. baumannii* and *K. pneumoniae* in oral samples resulted in a higher incidence of VAP of the same etiology during hospitalization (baseline: 100.0% vs. 22.2% and 80% vs. 9.1%, *p* = 0.003 and *p* = 0.013, respectively; follow-up 100.0% vs. 14.3%, 100.0% vs. 20%, *p* = 0.005 and *p* = 0.035).

The baseline and follow-up identification of *A. baumannii*, *K. pneumoniae* and *E. faecalis* in oral samples resulted in a higher incidence of any infection of the same etiology during hospitalization (baseline: 72.7% vs. 6.9%, 70% vs. 13.3%, and 41.2% vs. 13%, *p* < 0.001, *p* = 0.002 and *p* = 0.049, respectively; follow-up 85.7% vs. 4.8%, 80% vs. 17.4%, 50% vs. 5,6%, *p* < 0.001, *p* = 0.015 and *p* = 0.013).

There were no associations between BOAS scores, LOS and patients’ origin and the incidence of infection in the ICU.

In multiple logistic regression models for predictors of *A. baumannii*-HAI and *K. pneumoniae*-HAI, the only significant factors increasing the risk of infections were identification of *A. baumannii* and *K. pneumoniae* in the baseline oral sampling (ORs 35.1 and 16.2, respectively, Tables 5, 6, 7).

**Table 5:** Multiple logistic regression model for predictors of HAI of *K. pneumoniae* etiology

	*p*	OR	CI
EP versus SP	0.258	0.29	0.03–2.51
Baseline BOAS category 11–20 versus 0–10	0.557	1.97	0.21–18.90
Antibiotic before intubation (yes vs. no)	0.866	1.21	0.14–10.47
*K. pneumoniae* in baseline oral samples (yes vs. no)	0.005	16.24	2.34–112.80

N = 32

*OR*, odds ratio, *SP*, standard procedure, *EP*, extended procedure with tooth brushing, *BOAS*, Beck Oral Assessment Scale

**Table 6:** Multiple logistic regression model for predictors of HAI of *A. baumannii* etiology

	*p*	OR	CI
EP versus SP	0.506	0.445	0.04–4.82
Baseline BOAS category 11–20 versus 0–10	0.656	0.580	0.05–6.38
Antibiotic before intubation (yes vs. no)	0.452	0.346	0.02–5.50
*A. baumannii* in baseline oral samples (yes vs. no)	0.012	31.96	2.13–479.46

N = 31

*OR*, odds ratio, *SP*, standard procedure, *EP*, extended procedure with tooth brushing, *BOAS*, Beck Oral Assessment Scale

**Table 7:** Multiple logistic regression model for predictors of HAI of *E. faecalis* etiology

	*p*	OR	CI
EP versus SP	0.353	0.38	0.05–2.93
Baseline BOAS category 11–20 versus 0–10	0.375	0.45	0.08–2.61
Antibiotic before intubation (yes vs. no)	0.730	1.44	0.18–11.30
*E. faecalis* in baseline oral samples (yes vs. no)	0.243	2.82	0.49–16.08

N = 32

*OR*, odds ratio, *SP*, standard procedure, *EP*, extended procedure with tooth brushing, *BOAS*, Beck Oral Assessment Scale

### *Pulsed-field gel electrophoresis (PFGE, Fig. **1**)*

**Fig. 1:**
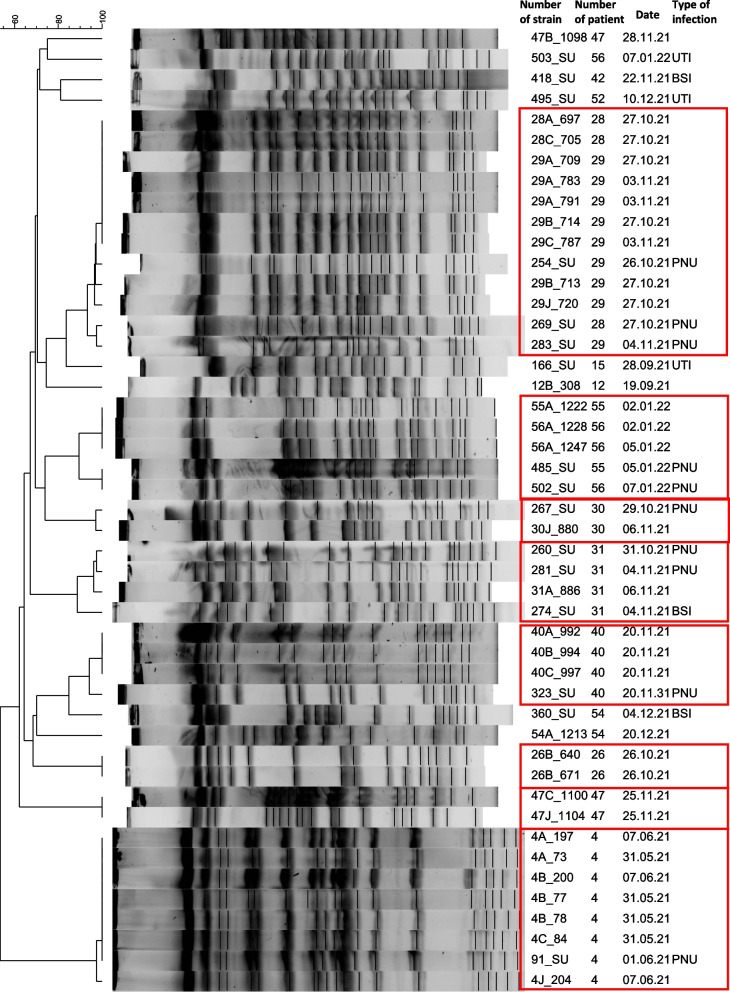
The results of pulsed-field gel electrophoresis (PFGE). *K. pneumoniae* strains. The results are presented for each patient with a number that they were assigned on recruitment (1–56). Rows ending in bloodstream infections (BSI), pneumonia (PNU), and urinary tract infections (UTI) represent HAI strains, and rows ending in blank space represent oral samples

The comparison of *K. pneumoniae* strains isolated from different habitats of the oral cavity showed that in 11 patients, these strains were identical. Only in one patient were two different strains identified in the oral cavity (patient 47).

Strains isolated from all PNU and one BSI case were the same as oral isolates in respective patients in 8 HAI cases (PNU: patients 4, 28, 29, 31 30, 40, 47; BSI: patient 31). In five HAI cases (UTI: patients 15, 52, 56; BSI: patients 42, 54), the strains isolated from HAIs were different from the oral isolates.

Additionally, in two pairs of patients (28 and 29 and 55 and 56), the presence of *K. pneumoniae* strains belonging to one clone was observed, both in the oral and HAI case strains.

## Discussion

In this study, we analyzed the dynamics of oral cultivable bacteriota and its association with HAIs, including VAP, among mechanically ventilated COVID-19 patients hospitalized in an ICU in the 7-day postintubation follow-up period. The oral bacteriota samples from both study periods revealed substantial dysbiosis, both qualitative and quantitative. During hospitalization, we observed a significant decrease in cultivable oral bacteriota diversity, with a high frequency of potentially pathogenic species, including *A. baumannii, K. pneumoniae* and *E. faecalis*, and a decreasing frequency of commensal *Streptococci* spp. strains. The incidence of HAIs was high, and their etiology correlated with the presence of *A. baumannii* and *K. pneumoniae* in the oral samples. The implemented procedure, which added tooth brushing to the standard oral care protocol, led to less frequent identification of *A. baumannii* and *E. faecali*s in oral samples; however, it was not sufficient for improving overall oral health or decreasing the risk of HAIs and mortality.

Both baseline and follow-up identification rates of selected bacterial species, such as *A. baumannii, K. pneumoniae, E. faecalis* and *E. coli,* in samples from the oral cavity were surprisingly high in our study population. During the observation period, those rates decreased but remained substantially high. The presence of *A. baumannii* and *K. pneumoniae* in oral samples was associated with HAIs of corresponding etiology. The presence of *A. baumannii* in oral samples was associated with VAP of the corresponding etiology. This finding is clinically significant, providing further evidence for a direct link between oral dysbiosis and VAP etiology [26]. Notably, for patients hospitalized in an ICU, the process is multifactorial. Salivary dysfunction due to medical procedures or medications and compromised oral care leading to accumulation of biofilm play an important role in the development of oral dysbiosis in this population. Additionally, the potential risk of cross-contamination between patients is listed among factors that can lead to a higher risk of dysbiotic oral bacteria reaching the lower respiratory tract [26]. Thus, providing proper oral hygiene can act as a protective measure against VAP [17, 19]. Using antiseptics, i.e., chlorhexidine, is a contested strategy [19]. Others include mechanical removal of dental biofilm, plaque and debris from the oral cavity to reduce the potentially pathogenic bacterial load and limit the risk of its aspiration to the airways [19].

In our study, we implemented an extended oral hygiene procedure that included tooth brushing as a measure of dental plaque control. The dental plaque of ventilated patients has been reported to present severe dysbiosis, with a high frequency of identification of *E. faecalis* and *E. coli* [27]. Oral dysbiosis of COVID-19 patients had been previously investigated and showed a high abundance of *Enterococcus spp.* and *E. coli* in oral samples from COVID-19 patients who did not require ICU hospitalization.

In the current study, patients undergoing the extended procedure with tooth brushing achieved better scores for the teeth subscale in the BOAS and, most importantly, had *A. baumannii* and *E. faecalis* less frequently identified in oral swabs. Unfortunately, it was not sufficient for improving overall oral health and decreasing the incidence of HAIs and mortality. One study reported similar observations, with lower BOAS scores and plaque index but no reduction in VAP incidence rates [28].

In our opinion, a lower identification rate of *A. baumannii*, a major cause of VAP in the ICU [29], in oral swabs is a first step toward effective VAP prevention. The fact that in our population it was not followed by a lower incidence of VAP could have resulted from significant dysbiosis and a high baseline number of patients with potentially pathogenic strains identified in the oral samples. Moreover, it is noteworthy that patients undergoing the extended procedure with tooth brushing had worse conditions of the oral cavity at baseline, thus probably limiting the efficacy of the intervention in comparison to patients with standard oral care. Analysis of PFGE results revealed that in some cases, PNU of *K. pneumoniae* etiology was diagnosed at the start of intubation (patients 4, 30, 40, 28, 29, 55, 56, Fig. 1), or additionally, the patients were subject to horizontal transmission of *K. pneumoniae* strains pre-ICU (patients 28, 29). In one case (patients 55 and 56 undergoing the standard procedure), PNU was diagnosed in the early postintubation period. Additionally, in one patient (56), UTIs of different *K. pneumoniae* strain etiologies were diagnosed. Finally, in one patient (31, undergoing the extended procedure with tooth brushing), PNU and BSI of the same *K. pneumoniae* strain etiology were recorded, suggesting BSI secondary to PNU and/or contamination outside the CVC (or PVC) or catheter hub by contact with hands. This implies that due to the high risk of oral contamination by potentially pathogenic microorganisms and early postintubation dysbiosis of the cultivable oral bacteriota, it may have been too late for the intervention to play a significant role in the prevention of VAP in our setting. It is possible that introducing proper pre-ICU oral care protocols could play some role in the prevention of HAIs in an ICU setting. In this case, patients in a clinical condition enabling proper comprehension and cooperation with the ward staff could take an active role in these measures. Finally, a single oral health care procedure may not be enough to assure good oral health, and such procedures should be bundled. A recent study of introducing oral bundle care in the place of chlorhexidine did not show a clear benefit for ventilated patients in the ICU [30]. Nevertheless, this issue requires further research.

The incidence of HAIs was high, affecting more than 70% of our study population, with bloodstream infections and VAP as the most frequent. The most common etiologies involved *K. pneumoniae* and *A. baumannii*.

HAIs in critical care are a major problem, with VAP as one of the most common infections that increases mortality and the length of hospitalization [20]. Multiple species are associated with VAP, especially *Pseudomonas aeruginosa*, *E. coli*, *K. pneumoniae* and *Acinetobacter* spp. [29]. The prevalence of multidrug resistant strains is high, and severe infections of this etiology are associated with mortality as high as 70% [31, 32].

In the population of COVID-19 patients, similar observations were made, with on average half those admitted to the ICU developing VAP (but some reports showed the rate of VAP to be as high as 86%), and ca. 43% mortality in those who developed VAP [33, 34]. In COVID-19 patients, *Enterobacterales* were reported to be the most common etiology of VAP (nearly 50%), but with mixed etiologies in up to 39% of patients [35]. The high incidence of VAP in COVID-19 patients is believed to result from a severe dysregulation of the immune system and hyperinflammatory reaction against SARS-CoV-2, leading to organ damage and further impairment of antimicrobial mechanisms [34].

Most previous studies investigating the relationship between SARS-CoV-2 virus, *K. pneumoniae* and *A. baumannii* focused mainly on the incidence of multidrug-resistant strains of those species in the ICU setting [36, 37]. One study showed similar results to ours, with COVID-19 increasing the risk of *A. baumannii* infections [38], with extremely high mortality rates in the elderly population.

There are some limitations to our study. The allocation protocol based on the fixed procedure performed in designated rooms did not assure sufficient control for confounders. Notably, analyses revealed that the patients undergoing the extended oral health care procedure with tooth brushing were in a more severe condition and had worse baseline oral health status. We believe that this may be one of the reasons for the lack of efficacy differences between the two investigated procedures but, importantly, it limited the risk of type I error.

Second, evidence of the dynamics of the oral microbiota among patients in a prone position is lacking. The prone position is a recommended method for mechanically ventilated patients. This affects transpulmonary pressure and achieves better blood oxygenation in patients with respiratory failure [39–42]. It has several advantages: the lungs are less compressed, gas exchange is more efficient, ventilator-induced injury risk is reduced and better drainage of secretions from diseased lungs is observed. However, we did not record the actual time spent in this position; thus, we were not able to ascertain its impact on oral bacteriota.

## Conclusions

Patients with a severe course of COVID-19 who are hospitalized in an ICU and require mechanical ventilation are at elevated risk of developing health care-associated infections. The most prevalent etiologies of HAI in this population were *E. faecalis, K. pneumoniae* and *A. baumannii*. Dysbiotic oral bacteriota is an important source of these pathogens. The introduction of tooth brushing in oral hygiene protocols in an ICU setting was effective in decreasing the extent of oral bacteriota dysbiosis; however, it did not reduce the risk of HAIs or mortality.

## Supplementary Information

**Additional file 1**. Appendix A1. Beck Oral Assessment Score (BOAS). Appendix A2. Oral care procedures.

## Data Availability

The datasets generated and/or analyzed during the current study are not publicly available due to institutional regulations but are available from the corresponding author upon reasonable request.

## Abbreviations

SD: Standard deviation
WHO: World Health Organization
ICU: Intensive care unit
BOAS: Beck oral assessment scale
DOT: Days of antibiotic therapy
HAI: Health care-associated infection
VAP: Ventilator-associated pneumonia
CFU: Colony forming unit
COVID-19: Coronavirus disease 2019
SARS-CoV-2: Severe acute respiratory syndrome coronavirus 2
ARDS: Acute respiratory distress syndrome
ERS: European Respiratory Society
ESICM: European Society of Intensive Care Medicine
ESCMID: European Society of Clinical Microbiology and Infectious Diseases
ALAT: Asociación Latinoamericana del Tórax
SOFA: Sequential organ failure assessment
GCF: Gingival crevicular fluid
PFGE: Pulsed-field gel electrophoresis
IQR: Interquartile range
OR: Odds ratio
