# Correction: Impact of tooth brushing on oral bacteriota and health care-associated infections among ventilated COVID-19 patients: an intervention study

**DOI:** 10.1186/s13756-023-01256-6

**Published:** 2023-05-29

**Authors:** Iwona Gregorczyk-Maga, Anna Pałka, Mateusz Fiema, Michal Kania, Anna Kujawska, Paweł Maga, Estera Jachowicz-Matczak, Dorota Romaniszyn, Agnieszka Chmielarczyk, Barbara Żółtowska, Jadwiga Wójkowska-Mach

**Affiliations:** 1 Institute of Dentistry, Faculty of Medicine, Jagiellonian University Medical College, Ul. Montelupich 4, 31-155, Kraków, Poland; 2 Microbiology Unit, University Hospital, Ul. Jakubowskiego 2, Kraków, 30-688 Poland; 3 Department of Endocrinology, University Hospital, Ul. Jakubowskiego 2, Kraków, 30-688 Poland; 4 Chair of Metabolic Diseases, Faculty of Medicine, Jagiellonian University Medical College, Ul. Jakubowskiego 2, Kraków, 30-688 Poland; 5 Doctoral School of Medicine and Health Sciences, Jagiellonian University Medical College, Ul. św. Anny 12, Kraków, 31-008 Poland; 6 Chair of Angiology, Faculty of Medicine, Jagiellonian University Medical College, Ul. Jakubowskiego 2, Kraków, 30-688 Poland; 7 Chair of Microbiology, Faculty of Medicine, Jagiellonian University Medical College, Czysta 18, 31-121, Kraków, Poland; 8 Center for Innovative Therapy, Clinical Research Coordination Center, University Hospital, Ul. Jakubowskiego 2, Kraków, 30-688 Poland

Antimicrobial Resistance & Infection Control (2023) 12:17

10.1186/s13756-023-01218-y

The original article [1] erroneously inverted each author’s name. The names have since been amended.
