# Supplementary material for: Impact of tooth brushing on oral bacteriota and health care-associated infections among ventilated COVID-19 patients: an intervention study
Source: Antimicrob Resist Infect Control. 2023 Mar 8;12:17. doi: 10.1186/s13756-023-01218-y (PMC9992909; doi:10.1186/s13756-023-01218-y)
Supplement: Supplementary file 1 — Additional file 1. Appendix A1. Beck Oral Assessment Score (BOAS). Appendix A2. Oral care procedures. [file 13756_2023_1218_MOESM1_ESM.docx]

**Appendix**

**Appendix A1**

| Beck Oral Assessment Score (BOAS), modified^a^ | | | | |
| --- | --- | --- | --- | --- |
|  | **Score** | | | |
| **Area** | **1** | **2** | **3** | **4** |
| Lips | Smooth, pink, moist, and intact | Slightly dry, red | Dry, swollen isolated blisters | Edematous, inflamed blisters |
|  | | | | |
| Gingiva and oral mucosa | Smooth, pink, moist, and intact | Pale, dry, isolated lesions | Swollen red | Very dry and edematous, inflamed |
|  | | | | |
| Tongue | Smooth, pink, moist, and intact | Dry, prominent papillae | Dry, swollen, tip and papillae are red with lesions | Very dry, edematous, engorged coating |
|  | | | | |
| Teeth | Clean no debris | Minimal debris | Moderate debris | Covered with debris |
|  | | | | |
| Saliva | Thin, watery plentiful | Increase in amount | Scanty and somewhat thicker | Thick and ropy, viscid or mucoid |
|  | | | | |
| Total Score^b^ | 5 No dysfunction | 6–10 Mild dysfunction | 11–15 Moderate dysfunction | 16–20 Severe dysfunction |
|  | | | | |
| Note: Provide moisture more often than oral care | Minimum care every 12 h | Minimum care every 8–12 | Minimum care every 8 h | Minimum care every 4 h |
| - BOAS 0–5 Perform an oral assessment once a day. Follow oral care as outlined in the systematic oral care procedure twice per day. - BOAS 6–10 Perform oral assessments twice a day. Moisten mouth/lips every 4 hours. Follow oral care as outlined in the systemic oral care procedure twice per day. - BOAS 11–15 Perform an oral assessment every shift (every 8–12 h). Follow oral care as outlined in the systematic oral care every shift. Use an ultrasoft toothbrush. Moisten lips and mouth every 2h. - BOAS 16–20 Perform an oral assessment every 4 hours. Follow oral care as outlined. If brushing not possible use soft gauze-wrapped finger. Moisten lips and mouth every 1 – 2 hours. | | | | |
| Modified from Beck and Ames  Ames NJ, Sulima P, Yates JM, McCullagh L, Gollins SL, Soeken K, Wallen GR. Effects of systematic oral care in critically ill patients: a multicenter study. Am J Crit Care. 2011 Sep;20(5):e103-14. doi: 10.4037/ajcc2011359. | | | | |

**Appendix A2**

The procedures used in the study were:

1. Standard oral care (cleaning and moisturizing of oral cavity, suction of excees fluid)
2. Extended oral care with teeth brushing (cleaning and moisturizing of oral cavity, teeth brushing, suction of excees fluid)

Standard mouth cleaning protocol (without brushing) included:

1. Preparation of sterile catheter (12F lub 14F), suction of excess fluid from the oral cavity.
2. Using Sage Suction Swab (Toothette®), soaking in Perox-A-Mint Solution and cleaning with circular movements of each mucosal site for 10 seconds:
   1. Right cheek mucosa and right upper quadrant (“przedsionek”)
   2. Left cheek mucosa and left upper quadrant
   3. Left lower buccal quadrant
   4. Right lower buccal quadrant
3. Moisturizing with Mouth Moisturizer /firma/ of oral cavity mucosa, tongue and lips.
4. Repeated suction of excess fluid from the oral cavity.

| **Activity** | **Equipment** | **Procedure** |
| --- | --- | --- |
| Preparation | Sterile suction cathether (12 or 14F) | Suction of excess fluid |
|  | | |
| Cleaning of the oral cavity | Sage Suction Swab Kit (SSS)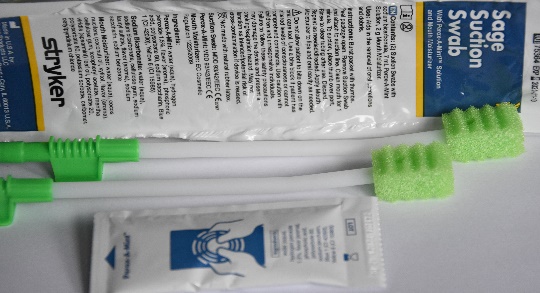  Perox-A-Mint mixture  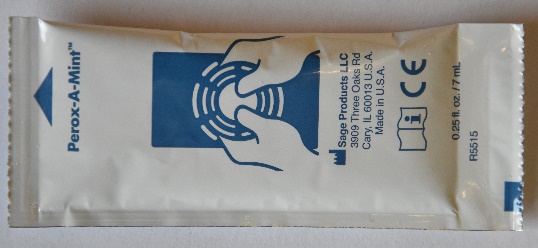 | 1. Soaking a sponge from z SSS kit using Perox-A-Mint 2. Cleaning with circular movements of each mucosal site for 10 seconds:    1. Right cheek mucosa and right upper quadrant (“przedsionek”)    2. Left cheek mucosa and left upper quadrant    3. Left lower buccal quadrant    4. Right lower buccal quadrant 3. suction of excess fluid |
|  | | |
| Moisturizing of the oral cavity | Sponge from SSS kit  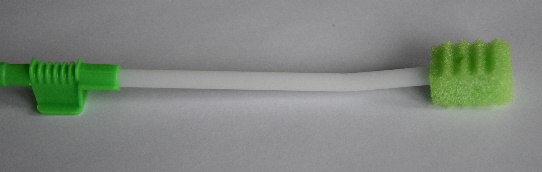  Moisturing mixture Mouth Moisturizer in sachet  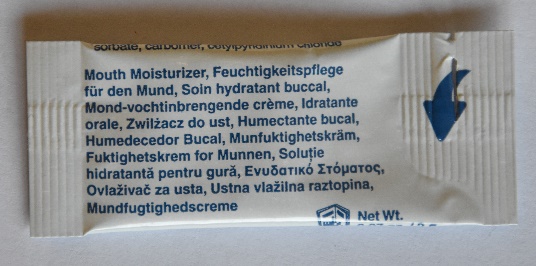 | 1. Moisturizing the sponge using Mouth Moisturizer 2. Moisturizing of of oral cavity mucosa, tongue and lips. |
|  | | |
| Suction of excess fluid from the oral cavity | Sterile suction catheter (12F or 14F) | 1. Suction of excess fluid |

Extended mouth cleaning protocol (without brushing) included:

1. Preparation of sterile catheter (12F lub 14F), suction of excess fluid from the oral cavity.
2. Teeth brushing (each teeth quadrant for 30) using Sage Untreated Suction Toothbrush (SUST) connected to suction unit, moistured with sterile water.
3. Using Sage Untreated Suction Toothbrush (SUST) moistured with sterile water and cleaning with circular movements of each mucosal site for 10 seconds:
   1. Right cheek mucosa and right upper quadrant (“przedsionek”)
   2. Left cheek mucosa and left upper quadrant
   3. Left lower buccal quadrant
   4. Right lower buccal quadrant
4. Moisturizing with Mouth Moisturizer /firma/ of oral cavity mucosa, tongue and lips.
5. Repeated suction of excess fluid from the oral cavity.

| **Activity** | **Equipment** | | **Procedure** |
| --- | --- | --- | --- |
| Preparation | Sterile suction cathether (12 or 14F) | | Suction of excess fluid |
|  | | | |
| Teeth brushing | Sage Untreated Suction Toothbrush kit (SUST)  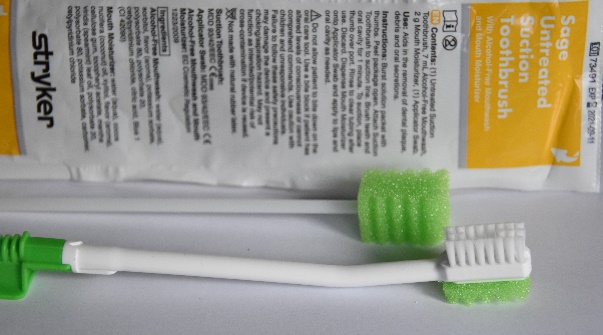 | | 1. Connecting the brush to the suction device 2. 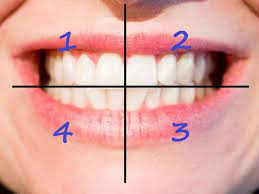Teeth brushing (each teeth quadrant for 30) using Sage Untreated Suction Toothbrush (SUST) connected to suction unit, moistured with sterile water. |
|  | | | |
| Cleaning of the oral cavity | Sage Untreated Suction Toothbrush kit (SUST)  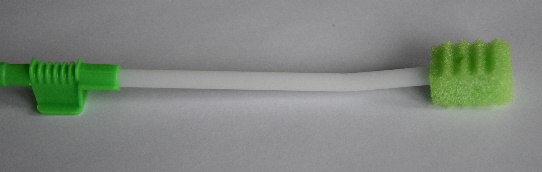 | 1. Moisturizing the sponge using sterile water 2. Using Sage Untreated Suction Toothbrush (SUST) moistured with sterile water and cleaning with circular movements of each mucosal site for 10 seconds:    1. Right cheek mucosa and right upper quadrant    2. Left cheek mucosa and left upper quadrant    3. Left lower buccal quadrant    4. Right lower buccal quadrant 3. Suction of excess fluid | |
|  | | | |
| Moisturizing of the oral cavity | Sponge from SSS kit  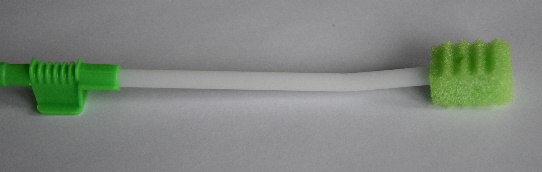  Moisturing mixture Mouth Moisturizer in sachet  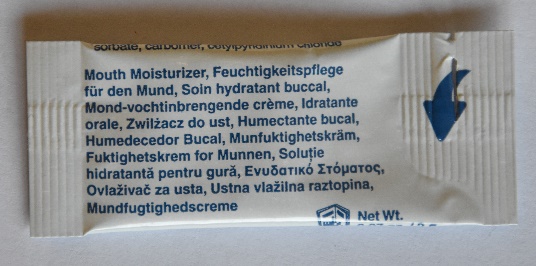 | | 1. Moisturizing the sponge using Mouth Moisturizer 2. Moisturizing of of oral cavity mucosa, tongue and lips. |
|  | | | |
| Suction of excess fluid from the oral cavity | Sterile suction catheter (12F or 14F) | | 1. Suction of excess fluid |
